# Making Connections: p53 and the Cathepsin Proteases as Co-Regulators of Cancer and Apoptosis

**DOI:** 10.3390/cancers12113476

**Published:** 2020-11-22

**Authors:** Surinder M. Soond, Lyudmila V. Savvateeva, Vladimir A. Makarov, Neonila V. Gorokhovets, Paul A. Townsend, Andrey A. Zamyatnin

**Affiliations:** 1 Institute of Molecular Medicine, Sechenov First Moscow State Medical University, Trubetskaya Str. 8-2, 119991 Moscow, Russia; ludmilaslv@yandex.ru (L.V.S.); known.sir@yandex.ru (V.A.M.); gorokhovets_n_v@staff.sechenov.ru (N.V.G.); 2 Division of Cancer Sciences and Manchester Cancer Research Centre, Faculty of Biology, Medicine and Health, University of Manchester, Manchester Academic Health Science Centre, and the NIHR Manchester Biomedical Research Centre, Manchester M13 9PL, UK; paul.townsend@manchester.ac.uk; 3 Belozersky Institute of Physico-Chemical Biology, Lomonosov Moscow State University, 119992 Moscow, Russia; 4 Department of Biotechnology, Sirius University of Science and Technology, 1 Olympic Ave, 354340 Sochi, Russia

**Keywords:** p53, cathepsin, cancer, MOMP, apoptosis, lysosomal membrane permeabilization

## Abstract

**Simple Summary:**

This article describes an emerging area of significant interest in cancer and cell death and the relationships shared by these through the p53 and cathepsin proteins. While it has been demonstrated that the p53 protein can directly induce the leakage of cathepsin proteases from the lysosome, directly triggering cell death, little is known about what factors set the threshold at which the lysosome can become permeabilized. It appears that the expression levels of cathepsin proteases may be central to this process, with some of them being transcriptionally regulated by p53. The consequences of such a mechanism have serious implications for lysosomal-mediated apoptosis and have significant input into the design of therapeutics and their strategic use. In this review, we highlight the importance of extending such findings to other cathepsin family members and the need to assess the roles of p53 isoforms and mutants in furthering this mechanism.

**Abstract:**

While viewed as the “guardian of the genome”, the importance of the tumor suppressor p53 protein has increasingly gained ever more recognition in modulating additional modes of action related to cell death. Slowly but surely, its importance has evolved from a mutated genetic locus heavily implicated in a wide array of cancer types to modulating lysosomal-mediated cell death either directly or indirectly through the transcriptional regulation of the key signal transduction pathway intermediates involved in this. As an important step in determining the fate of cells in response to cytotoxicity or during stress response, lysosomal-mediated cell death has also become strongly interwoven with the key components that give the lysosome functionality in the form of the cathepsin proteases. While a number of articles have been published highlighting the independent input of p53 or cathepsins to cellular homeostasis and disease progression, one key area that warrants further focus is the regulatory relationship that p53 and its isoforms share with such proteases in regulating lysosomal-mediated cell death. Herein, we review recent developments that have shaped this relationship and highlight key areas that need further exploration to aid novel therapeutic design and intervention strategies.

## 1. Introduction

As a nuclear transcription factor discovered as far back as 1979, the functional role of the tumor suppressor protein TP53 (p53) has evolved centrally in the regulation of DNA repair, cell cycle, and apoptosis [1,2]. Through such studies, p53 has revealed itself to be justifiably labelled as the “guardian of the genome” [3]. Mechanistically, this originates from its ability to be activated upon cellular stress and orchestrate the cell’s DNA damage response and, in doing so, helping to maintain genome integrity [4]. Consequently, the mutation of the functional p53 gene can give rise to the accumulation of a variety of cancer types [5] through its deregulated modulation of cellular senescence and apoptotic death signaling pathways [6]. Over the years, this aspect of p53 activity has unveiled a number of interesting phenomena that determine cell fate under normal conditions and throughout disease onset or progression. Moreover, through the identification and characterization of a number of *TP53* somatic mutations and its isoform proteins, our understanding of the molecular events that give rise to deregulated p53 activity with such serious and physiologically relevant effects has vastly improved [7,8].

**Figure 1:**
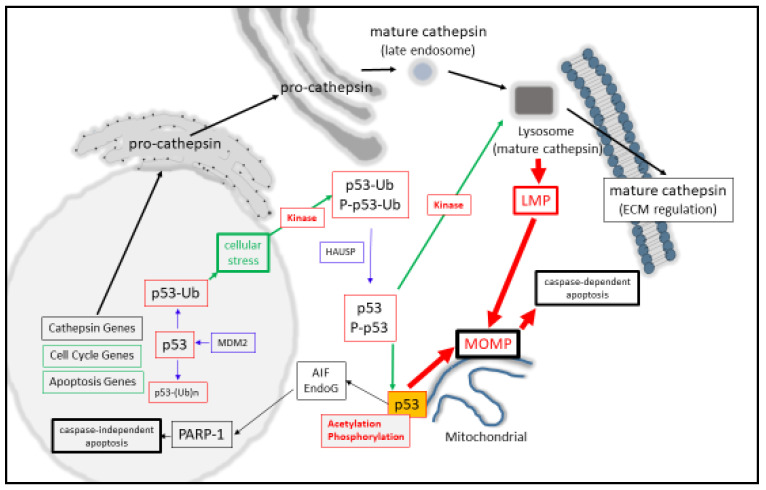
p53 regulates lysosomal- and mitochondrial-mediated apoptosis.

At the molecular level, monomeric-p53 is composed of 393 amino acids and is active as a homo-tetramer protein [9,10], which can be post-translationally modified in a variety of ways that modulate its activity [11,12,13,14]. One key regulatory step that keeps nuclear p53 in its inactive state is through minimizing its protein stability [15,16] through its poly-ubiquitination (and proteasomal degradation) by MDM2 in a negative auto-regulatory feedback manner [17]. Moreover, mono-ubiquitination can also behave as a signal for p53 to be cytoplasmically [18,19] or mitochondrially translocated [20], where it can undergo de-ubiquitination by herpesvirus-associated ubiquitin-specific protease (HAUSP) upon its arrival [21]. Another post-translational modification of p53 is protein acetylation (at Lysine-120), and this form of acetylated p53 is found at the mitochondrial outer membrane after DNA damage induction and displaces Mcl-1 from BAK [22] and positively regulates apoptosis. Simultaneously, the activation of p53 can also occur through DNA damage-induced phosphorylation (by HIPK2, for example) at Serine-46 as an important event for mitochondrial localization [23,24], and can cause the direct induction of Mitochondrial Outer Membrane Permeabilization (MOMP, [25,26]). Phosphorylation can also have the effect of stabilizing nuclear p53 levels [14], permitting it to modulate target gene expression, as seen with the cell cycle regulator p21 [27], either directly or with the aid of co-activators [28] (Figure 1).

Nuclear p53 is stabilized upon cellular stress or DNA damage (green box) and can regulate cell cycle and apoptosis (orange boxes) and translocate to the cytoplasm as a mono-ubiquitinated species, where it can become phosphorylated and induce lysosomal membrane permeabilization (LMP). The released cathepsin proteases induce mitochondrial outer membrane permeabilization (MOMP) and caspase-dependent apoptosis. Mono-ubiquitinated p53 can also be deubiquitinated by HAUSP, allowing p53, phosphor-p53 (P-p53), or acetylated forms of it to reside in the outer membrane of the mitochondrion and induce MOMP or the release of Apoptosis Inducing Factor (AIF) and EndoG, which mediate caspase-independent apoptosis through PARP-1 activity. Cathepsin genes (black box) are transcribed, translated, enter the secretory pathway, and are ultimately secreted for Extracellular Matrix (ECM) regulation or traffic to the lysosome as mature cathepsin proteases. Lysosomal cathepsins can be released into the cytoplasm through LMP, leading to MOMP and caspase activation.

One mechanism proposed for the nuclear retention of p53 is through its tetramerization, which results in its nuclear export signal being masked [29]. Through such important regulatory events, p53 can induce reversible cell cycle arrest [30], which permits enough time for DNA repair mechanisms to be completed, particularly at low levels of DNA damage [31]. However, upon extensive DNA damage cell cycle arrest is prolonged and cells also undergo caspase-independent apoptosis [32,33,34] prior to their clearance. In the former, this can occur through the p53-mediated up-regulation of *APAF-1* [35,36], *NOXA* [37], *PUMA* [38,39], *BAX* [40,41], and *BIM* [42,43] and through suppressing *BCL-2* [44,45,46], *BCL-XL* [46,47,48], and *MCL-1* gene expression [49,50,51,52]. Mechanistically, mitochondrially located active p53 can bind and inhibit the activities of Bcl-2 and Bcl-xL proteins while enhancing the release of mitochondrial AIF and EndoG [53,54,55] upon the apoptotic stimulation of cells, causing these proteins to translocate to the nucleus, where they mediate large-scale chromatin fragmentation [56] in a PARP-1-dependent manner (as reviewed recently [57]).

Intimately connected with cell death is the lysosome. While initially an organelle that could induce the demise of cells through its leakage or rupture (as a “suicide bag” [58]), the protein factors that have been identified to play a key role in relaying such an effect are the cathepsin proteases [59]. The cathepsin protease family can be subdivide into aspartic (D, E)-, serine (A, G)-, and cysteine (B, C, F, H, K, L, O, S, V, Z/X, W) proteases subtypes, the expressions of which have been intensely researched in the context of normal cellular homeostasis and disease progression [59,60]. Cysteine cathepsins have gained considerable attention due to their ability to be auto-activated, being catalytically active at acidic and neutral pH and resident within other intra-cellular compartments, including the cytoplasm, mitochondrion, and nucleus, where they have been reported to positively modulate a number of key pathways involved in cell cycle regulation and cellular trans-differentiation [61].

Briefly, lysosomal-derived cell death can take a number of forms when viewed mechanistically at the molecular level. While the leakage of cathepsins into the cytoplasm can activate the classic apoptosis pathway through BID-mediated MOMP [62,63,64], they can also cleave and release mitochondrial AIF [65,66,67], which is an important step in PARP-mediated caspase-independent cell death [68,69] (Figure 1).

In certain cancer cells, cathepsin proteases can also be found at the cells surface, where they can modulate the extracellular matrix during tumor progression [59]. For example, enhanced cathepsin D expression was observed in the invasive front of Oesophageal Squamous Cell Carcinoma tumors (in comparison to the cancer nests [70]) and is a common feature in gastric carcinoma [71], oral carcinoma [72], and colorectal carcinoma [73]. Here, it is interesting to note that a shift in localization of cathepsin D from the lysosomes to the cells surface can occur, as reported in models of oral carcinoma progression [72] (Figure 1).

How the lysosome and its constituent protein factors might co-regulate p53-dependent (or independent) cell death [70] has been a very active area of research over the years. Here, the expression profiling of p53 with cathepsins or other marker proteins of interest (such as mTOR and IGF-R1 [74]) for diagnostic or prognostic purposes within the clinic and offering mechanistic insights into how the p53 protein may modulate key subcellular organelles (such as the mitochondria or lysosome) and the signaling pathways arising therefrom are good examples. Consequently, some very promising findings have emerged, some of which have been extended to therapeutic development and evaluation. For example, p53 is also recognized as a stress sensor protein that can respond to a number of signaling cues arising from viral infection, starvation, oxidative stress, and the mammalian Target of Rapamycin (mTOR) pathway (reviewed in [75]). Here, MDM2 activity can be accelerated upon its phosphorylation by AKT kinase, and when the PI3K/mTOR pathway was targeted using inhibitors such as DS-7423, an increase in the p53 levels and the apoptosis of ovarian cancer cells were observed [76,77]. Such promising therapeutic approaches have also been extended through the use of nanoparticle delivery methods (reviewed by [78]).

From a broader perspective, while the signaling transduction pathways underpinning p53 effects are emerging with greater clarity, many aspects of the molecular regulatory mechanisms defined by these do present additional (and fundamental) questions. One key aspect of such a paradigm is the relationship shared by p53 expression and the cathepsin proteases in modulating the intrinsic arm of the apoptosis pathway through lysosomal-mediated cell death. Clearly, this aspect of p53-induced cell death does not appear to be as developed as the p53-mediated mitochondrial mediated cell death pathway.

In this review article, we focus on the importance attributed to the role that p53 plays in mediating lysosomal-mediated cell death over recent years. We extend these findings through emphasizing how p53 (or its isoform proteins) may modulate cathepsin protease expression as a potentially important mediator of the threshold at which Lysosomal Membrane Permeabilization (LMP) occurs, which consequently gives rise to lysosomal leakage and the activation of apoptosis (Figure 1). To support such a notion, we also present preliminary bioinformatics-based analyses, which addresses the potential of p53 in modulating cathepsin gene expression as a key area of research in order to further define this relationship.

### 1.1. p53 Transcription and Functionality: An Update

Genetically, the p53 protein is encoded by 10 exons through a mature mRNA transcript spanning 2512 bp, which can translate from a number of translation start sites or with a number of alternatively spliced variants of the p53 pre-mRNA, giving rise to a number of isoform proteins [79,80]. The importance of the p53 protein has gained considerable momentum following the discovery that the p53 gene is found to be mutated (through deletions and point mutations) in over 50% of cancer types [2,81], which can give rise to the onset of disease [82]. Alternatively (and paradoxically), p53 over-expression can therefore be found in a high number of cancer types, albeit in its mutated form in most instances [2,83,84]. Based on the growing numbers of such mutated gene products, they can be classified as “contact” or “structural” mutations (based on alterations in their DNA-binding ability), such as the contact mutants R248W, R273C, and R73C [83] and the structural mutants R175H [85,86], R282W, [87], and Y220C [88]. Both groups give rise to loss of function. Conversely, a number of mutations can give rise to a gain of function through their ability to permit the aggregation of the p53 protein while enhancing their regulatory interactions with other transcription factors [89,90].

In offering greater context, paralleled progress has also been made in the key area of defining p53-mediated miRNA regulation (or vice versa). For example, p53 can regulate the processing and maturation of miRNA [91], while p53 itself can also be regulated by miRNA [91] or indirectly through MDM2-specific modulation by miRNA expression [92]. In keeping with p53-mediated transcription, another interesting research area of growth has been the area of defining (with greater clarity) the p53 consensus sites (and their structural requirements) present upstream of its target genes [93,94]. Herein, a number of excellent studies have emerged, which have given rise to the characterization of the p53 DNA-binding consensus sequence (see Section 1.6 below) and what significance the structure of the DNA harboring this sequence can offer in p53-DNA recognition. Of equal importance has been the discovery that the p53 gene can also bind the p63 and p73 consensus sequences and suppress gene expression [95,96]. While such findings do offer a number of important findings insofar as the role that the p53 protein may play in regulating cell death (either indirectly or directly), it also diversifies the role that p53 may play in the regulation of genes once thought to be regulated exclusively by transcription factors p63 and p73 alone.

### 1.2. Lysosomal Membrane Permeabilization—A Key Event in Cell Death

Throughout the lysosomal-mediated induction of cell death, it is now accepted that the causative agents of this event are the cathepsin proteases, acting through BID cleavage and BAX-mediated MOMP [62,63,64]. Mechanistically, the loss of lysosomal cathepsin proteases to the cytoplasm (through Lysosomal Leakage, LL) can be brought about by a number of mechanisms that act through inducing LMP. Of importance may be how the lysosome changes its functional characteristics during disease progression and how this may alter its ability to undergo LMP to varying degrees. As an example, some cancer cells have been reported to possess larger (and more fragile) lysosomes and consequently be more sensitive to the LL-inducing effects of LMP agents in comparison to normal cells [97]. Such lysosomorphic agents include imidazole [98], Sphingosine [99], a Riccardin A-derivative [100], and Siramesine [101,102]. Alternatively, the inhibition of signal transduction intermediates, such as Sphingokinase 1 (SPK1), can also induce LMP, with a loss of intra-lysosomal sphingosine-1-phosphate, thus giving rise to abnormal lysosomes [103].

In view of lysosomes inducing cell death [104], protecting lysosomal integrity therefore takes on paramount importance [105] and can be mediated by Heat Shock Proteins (HSP) HSP70 and HSP90, anti-apoptosis proteins Mcl-1 and Bcl-xL, anti-oxidation proteins (such as super oxide dismutase, glutathione peroxidases and catalases), and the Lysosomal-Associated Membrane Proteins (LAMP) 1 and 2 [105].

Of importance are also the effects that cathepsin proteases can inflict on LMP- and LL-regulation. For example, cathepsin B is required for TNF-α-mediated LMP [106], while TNF-R1 internalization can also induce LMP in a cathepsin D-dependent manner [107]. Moreover, the localization of cathepsin B within nuclei can also reduce lysosomal breakdown [108].

When taken with SPK1 as being a cathepsin B substrate [109,110] and the LAMP1/2 levels being reduced through cleavage by cathepsin B overexpression [111,112], such findings indeed support how the threshold of LMP can be altered in a cathepsin expression-dependent manner. This has clear relevance in the context of justifying the development of lysosomorphic agents for therapeutic purposes, which has already been given effect through their successful targeting of the lysosome in a number of cancer types, such as colon cancer [111,113], breast cancer [101,114], cervical cancer [114], acute myeloid leukemia [115], prostate cancer [114], lung cancer [116,117,118], ovarian cancer [114], and skin cancer [116].

### 1.3. p53 As a Co-Regulator of Mitochondrial and Lysosomal Mediated Cell Death

Synonymous with activating LMP, a broad spectrum of agents such as reactive oxygen species, DNA damaging agents, heavy metals, ischemia, and inflammation can also result in the activation of p53 [119]. The contributing factors that have helped to resolve the importance of p53 for inducing death have arisen from the question of how effectively cells die in a p53-dependent manner. Here, p53-mediated death can be arrived at through the up-regulated expression of BAX [41], APAF-1 [35,36], NOXA [37], PUMA [38,39], BIM [42,43], Bcl-2 suppression [44], Bcl-xL [46,47,48], and Mcl-1 suppression [49,50]. Consequently, MOMP [25] can be directly induced by activated P-p53 [23], through activating BAX or BAK [25,120,121], or through displacing anti-apoptotic Bcl-2 proteins from their pro-apoptotic counterparts [25,120,122]. However, a second contributing mechanism for this may also be involved through the actions of p53 mediating LMP directly as the initiating step of lysosomal-mediated cell death through MOMP induction (Figure 1). Such a suggestion comes from p53 directly inducing LMP in myeloid leukemia cells [123] and TNF-α-treated fibrosarcoma cells [124]. Herein, LMP activation strongly depends upon the localization of phosphor-Ser15-p53 to the lysosomal membrane [124], which could destabilize the lysosome and induce apoptosis in a lysosomal-mitochondrial linked pathway [123].

Based collectively on p53 and cathepsin being synonymous in their roles in destabilizing the lysosome, it appears that both of them therefore may have significant input into how effectively lysosomes are predisposed to LMP or LL. While high levels of cytoplasmic p53 may cause LMP, during low levels of p53 LMP inducibility may possibly be compensated for by the relatively high level of expression of cathepsin proteases. Clearly, such a mechanism requires there to be some regulatory relationship between the cathepsin proteases and p53 at the transcriptional, translational, or post-translational level.

### 1.4. p53 Activation and Cathepsin Protein Expression

As central factors that are heavily involved in mediating LMP, clearly one question that arises from our viewpoint is whether there is a direct regulatory relationship between the p53 and cathepsin proteins. To date, vital information addressing this has been arrived at through the analysis of p53 and cathepsin protein or mRNA expression levels in p53-positive or -negative cells, or through their evaluation as co-expressed diagnostic or prognostic markers, either in tissue samples or from serum [125]. Here, of central importance may be the p53 consensus binding sequence PuPuPuC(A/T)(A/T)GPyPyPy, which can be present in duplicate and separated by a 13 bp spacer and has the core consensus sequence C(A/T)(A/T)G. Through a deletion analysis of this sequence, p53 can bind the full site, half site, and 1.5 sites [10,126], and activate them all in reporter gene assays [126,127,128,129].

Being mindful of the aforementioned, a number of excellent studies have reported a very strong correlation between p53 expression and the regulation of some cathepsin genes. For example, Wu et al. (1998) demonstrated cathepsin D was expressed in a p53-dependent manner in U1752, Pa1, and ML1 leukemia cells following Adriamycin stimulation. Additionally, p53 could bind two p53 consensus sites within the cathepsin D promoter in vitro and direct luciferase-reporter gene activation in cells. Enhanced cathepsin D expression also offered cells a greater resistance to death following Etopiside treatments [130].

More recently, the anti-tumor effects of WIN55-212-2 in glioblastoma cell lines were evaluated and a positive relationship observed between DNA-damage induced *mut*-p53 and cathepsin D protein levels [131].

In the instance of cathepsin L, Katara et al. (2010) identified p53 as a positive binding factor to the cathepsin L promoter and *mut*-p53 reported to positively regulate cathepsin L expression in glioblastoma cells [132]. In a similar cellular context, the cathepin L expression was positively correlated with the *mut*-p53 expression in glioblastoma U251 cells treated with Ionizing Radiation (IR) and the inhibition of which (through cathepsin L inhibition) sensitized cells to IR-induced apoptosis [133]. In confirmation of such findings, *mut*-p53 also enhanced cathepsin L expression upon IR-induced EMT of NSCLC cells [134]. Lastly, cathepsins B and D activity could be enhanced in the rat hippocampus during IR therapy for 12 h, which correlated with enhanced WT-p53 proteins levels and which could be reversed upon treatment of cells with Pifithrin-α [135]. Supportingly, Xin et al. (2019) reported that cathepsin S and K mRNA expression was induced in line with p53 protein expression during chronic oxidative stress conditions in mouse aortic tissues [136].

Based on these collective findings, clearly p53 has some direct input into cathepsins D, L (and likely B) transcriptional and/or protein regulation. Herein, while some studies have explored the role of WT-p53, others had focused on *mut*-p53, and which generally emphasize a growing connection between p53 and cathepsin expression with EMT, oxidative stress, and chemotherapeutic resistance or sensitivity (Table 1).

### 1.5. p53 and Cathepsin Expression in Tumor Samples: A Clinically Relevant Relationship

The development of cell line models in establishing a clear-cut relationship between p53 activation and cathepsin gene regulation have certainly laid a number of very solid foundations in exploring this relationship further (as outlined in Section 1.4). Consequently, such findings have been extended into a clinical setting and which have yielded some very encouraging outcomes with some cathepsins showing a clear prognostic relationship with p53 expression in a cell-type context manner. Moreover, this relationship has also unveiled a more detailed picture with regards to which cathepsins and *mut*-p53 derivatives are co-expressed in relation to stage-specific tumor progression. Collectively, such approaches have offered greater insight into defining the physiological value of such a relationship with greater clarity. Therefore, in the following section, we extend and detail these findings to highlight the clinical relevance of the findings highlighted in Table 1, with a view to outlining consistent prognostic and diagnostic trends that may be emerging from the assessment of cathepsins and p53 expression from patient samples when viewed from a clinical perspective.

As far back as 1997, cathepsin D expression was reported as being upregulated in 47% of the 152 lung adenocarcinoma patient samples with good prognostic predictability using Immunohistochemistry (IHC) staining and analysis. Here, Higashiyama et al. (1997) predicted the outcomes for stage 1 patients who tested positive for stromal cell cathepsin D expression as having a poor prognosis [137]. Shortly thereafter, another independent study compared small cell carcinoma samples of the lung taken from short-term survivors with prolonged survivors for cathepsin B and D expression using IHC analysis. Here, cathepsin B was absent in 23% of prolonged survivors and cathepsin D absent from 87% [138]. Similarly, in pursuit of an accurate diagnostic and prognostic assay, an alternative approach utilizing Broncheoalveolar Lavage (BAL) fluid taken from 50 resectable NSCLC patients and a molecular diagnostic PCR-based screening approach identified 28 patients expressing mutant-p53 in stages I-IIIA of tumor progression [139]. Such studies (individually and collectively) validated the potential of utilizing cathepsin expression analysis within a clinical context and highlight alternative approaches that can be developed for robust assay development.

Based on the importance held by the differential expression of the aforementioned proteins and cancer progression, a number of very good studies were to follow addressing the use of these two proteins as potential diagnostic (or prognostic) markers for a number of cancer types. For example, Lazaris et al. (2000) analysed 64 patient samples for Squamous Cell Carcinoma (SCC) of the larynx using IHC staining, from which 57.8% showed the accumulation of nuclear p53. Importantly, simultaneous cathepsin D expression was localized to the tumor parenchymal and stromal cells in 31.25% and 37.5% (respectively) and was understood as being a useful indicator for defining patient subgroups that showed variations in relapse-free survival. This was based upon subgroups containing subglottic- or transglottic- tumors, where patients with tumor-positive lymph nodes showed positive for cathepsin D expression and were classed as being at a higher risk for relapse [140].

In the context of Inflammatory Breast Cancer (IBC), Aziz et al. (2001) analyzed the expression profiling relationships between p53 and cathepsin D using 40 patient samples of grade II and III IBC origins. Through the use of IHC and flow cytometry analysis, *mut*-p53 expression was observed in 70% of such patients and cathepsin D expression was detected in 30% of these [125]. Herein, the prognosis for *mut*-p53 expressing patients was seen as being significantly poor. Such findings were in strong contrast to an earlier report where 100% of the 22 IBC samples stained for cathepsin D expression tested positive [141].

Through testing a larger cohort of patients, Ikeguchi et al. (2000) reported high levels of cathepsin D and p53 expression in 49% and 46% of the 154 esophageal SCC samples tested, respectively. Here, the cathepsin D expression was correlated with invasiveness and poor prognosis. More importantly, p53 and cathepsin D co-expression was observed in 29.2% of the samples tested, with cathepsin D expression not showing as an independent prognostic factor [70].

While Aziz et al. (2001) reported the encouraging detection of cathepsin D or p53 expression for IBC and disease progression, in the context of pregnancy/lactation associated breast carcinoma (PAC), no significant differences in survival between controls and p53 or cathepsin D expression were observed in the 379 samples tested using IHC and flow cytometry analysis [142]. However, in a later study Ioachim et al. (2003) assessed 134 primary invasive BC patients, where extensive stromal cathepsin D expression correlated positively with p53 expression using an IHC approach [143]. Here, stromal cathepsin D was associated with a poor patient outcome and unexpectedly exhibited a marginal relationship with Estrogen Receptor (ER) expression [144].

More recently, Losch et al. (2004) tested a number ovarian tumors using IHC staining for p53 and cathepsin D expression [145]. Here, 43 low malignant potential and 80 invasive tumor samples were analysed with 65.1% and 43.7% showing positive for cathepsin D expression, respectively. Cathepsin D expression showed a negative relationship with stromal p53 and could be used as an independent prognostic factor for disease-free survival in patients with invasive ovarian cancer (OC).

In the context of Colorectal Cancer (CRC) and the IHC analysis of 266 patient samples, 38.7% and 60.9% of these were seen as cathepsin D and p53 positive, respectively [146]. Herein, cathepsin D expression was correlated with reduced cancer-free survival and as an independent factor for poor prognosis. Moreover, p53 and cathepsin D expression could also be correlated with distant metastases.

While the focus of prognostic and diagnostic assays had revolved around cathepsin D and p53, Sun et al. (2016) evaluated the use of cathepsin D along with cathepsins-B, -G, -K, -L, and -V in 188 BC tissue samples using IHC staining [147]. Herein, cathepsins V and D expression were seen to be associated with BC metastases, whereas cathepsins B and D expression were associated with poor disease-free survival. In partial support of such findings, Guerra et al. (2016) analyzed 217 samples for p53 and cathepsin D expression and the expression of both having significant association with BC relapse [148]. Importantly, the insertion or deletion mutations of the p53 gene were identified as potentially new prognostic markers for BC relapse and local invasiveness.

To summarize, the use of cathepsin protease expression as a diagnostic or prognostic tool has shown good promise over the years (Table 2). While the most clinically studied cathepsins in cancer progression have revolved around cathepsin D, progress has indeed broadened towards looking at other cathepsins for their usefulness in such assays, permitting their importance in cancer progression to come into focus [147]. Additionally, previous assays have focused on detecting the expression of WT-p53 or highly expressed *mut*-p53, and the outcomes have often been seen to be quite inconsistent and therefore difficult to evaluate. As briefly highlighted above, this may be due to variations in cohort sizes or due to the variable nature of the samples tested, but nevertheless some very encouraging findings have emerged with regards to where (and to what extent) some cathepsins are expressed in tumor progression and what relationships they share with other prognostic protein markers. In the instance of p53 expression, while some studies (in CRC, for example) have suggested that p53 expression is associated with a poor prognosis [149], others have stated the contrary [150] or stated that no such relationship between p53 and survival exists [151]. While the reasons for these may yet be waiting to be discovered, extending such studies to incorporate the detection of isoform p53 proteins may offer some insight, particularly as the use of IHC can offer limitations in what type of p53 protein species can be detected (and to what extent) with absolute certainty. Nevertheless, this aspect of testing tissue samples may be starting to gain traction based on the excellent study published by Guerra et al. (2016) [148], particularly as researchers look towards taking a more Integrative approach in validating the significance of the biomarkers they are assaying for [152].

### 1.6. Cathepsin Genes As Potential Targets for p53

Clearly in some instances, p53 can have the potential to directly regulate cathepsin expression at the transcriptional level. Whether this results in up- or down-regulation of expression, is WT-p53, *mut*-p53 or cell context-dependent, are questions that come to mind and offer greater justification in looking at this potential regulatory relationship further.

To help identify and confirm the existence of p53-specific consensus sequences, which may or may not regulate gene transcription, a genome-wide chromatin immunoprecipitation analysis using the high-throughput sequencing of p53-specific DNA fragments was adopted. Briefly, T-lymphocytes were stimulated with Doxorubicin, Nutlin-3, or carrier for 24 h and p53-bound chromatin fragments were eluted, cloned, and had their DNA sequence determined. From this study, 770,000 potential p53 target sites which led to the development of the Human p53 Binding and Expression Resource (BAER) database were successfully identified [153]. Surprisingly, only 23% (12,885/54,947) contained the full p53 duplicate- or half-p53-like consensus sequence site sequences that could bind active p53. Under unstimulated control conditions, 68% (1727/2532) contained a p53-like motif, of which 43% were present within (or near) the 5′ region of the transcription start site, with 20% in intragenic and 37% in intergenic regions. Moreover, such studies demonstrated that the most common of the p53 consensus sequence occurs without a spacer and encodes only half of the duplicate p53 consensus site [153]. Upon scrutinizing the BAER database for cathepsin genes that may harbor potential p53 binding site half sequences, the existence of such sequences and their prevalence in a significant number of genes either as p53 consensus sites or as sequences that can bind p53 and which are unrelated to the p53 consensus sequence was revealed (Table 3). Additionally, such sequences were found to be present within promoter sequences and within the coding- and non-coding regions of the genes. Likewise, upon using the Matinspector platform [154] to search the -3 kb gene sequences of the cysteine cathepsin proteases and cathepsin D (as taken from the Ensembl database), our findings also confirmed the presence of p53 half-sequences (Table 4). Collectively, such preliminary findings do suggest that a stronger link may exist between p53 and the transcriptional regulation of some members of the cathepsin protease family (than originally thought), and the experimental confirmation of these theoretical findings is eagerly awaited.

## 2. Future Directions

Cathepsin expression levels may set the threshold at which LMP occurs in response to lysosomorphic agents or otherwise. P53 in all its mutant isoforms may contribute to this based on how it regulates cathepsin gene transcription and may be a relevant mechanism for controlling LMP based on the status of p53 expression. Of importance here is the direct relationship between p53 and cathepsin expression. On one hand, p53 can enhance the gene expression of some cathepsins, which promote tumor progression through modulating the ECM and give rise to cells that contain abnormal lysosomes. On the other hand, cells lacking p53 (as in most tumors) would be expected to have diminished levels of cathepsin expression, which does not appear to be the general case in tumor cells (as cathepsins are generally seen to be overexpressed in a dysregulated manner). Such a paradox clearly highlights the need for additional studies that address the dynamics of the p53-mediated transcriptional regulation of cathepsin gene expression. Such studies clearly also need to incorporate the further characterization of p53 isoforms (or their mutant derivatives), epigenetic changes to the cathepsin promoter regions, or even their responsiveness to the isoform proteins of the p63 and p73 transcription factors.

While mitochondrial-mediated apoptosis (through MOMP and AIF release) is well characterized, additional studies may also need to be performed to delineate how the lysosome may be regulated by p53 in a similar manner. Clearly, the questions that need to be addressed may include asking what p53 post-translational modifications are critical for this and whether the p53 isoforms or mutant derivatives have any influence on modulating LMP.

Clearly, the aforementioned research avenues emphasize the complexity of the interwoven signaling mechanisms that are emerging in this area of research and highlight, with greater clarity, the key factors that need to be given extra weight moving forwards.

## 3. Conclusions

The connectivity between p53 and the variety of death-inducing pathways it is involved in is indeed coming into greater focus based on the diversity of reports published over the recent years. While originally believed to be a gene product that can suppress tumor formation through the regulation of the cell cycle and apoptotic gene products, the role of p53 has emerged as being quite diverse, in that it also has great significance in lysosomal-mediated cell death. Clearly, the prelude to such an event revolves around lysosome functionality and its predisposition to undergo permeabilization in normal and diseased tissues and whether the power of this organelle can be harnessed sufficiently well to trigger the demise of cancer cells. Two key components that may be of relative importance in this “regulatory switch” are p53 and the cathepsin proteases, the expression of which can be deregulated during disease progression and which are firmly interconnected at the transcriptional and protein level. To date, sufficient interest has indeed developed in these key areas to explore whether *mut*-p53 protein species or the alternatively spliced isoform variants of p53 modulate the cathepsin proteases at the transcriptional level or regulate LMP.

## Figures and Tables

**Table 1:** Wild Type (WT) or mutant (mut) p53 with cathepsin protein expression levels can be positively correlated in a variety of cancer cell lines and types.

p53 Expression	Cathepsin	Cells	Reference
WT-p53 increased	D	Lung, Ovarian, Leukemia cells	[130]
*mut*-p53 increased	L	Glioblastoma	[132]
WT-p53 increased	B	Rat hippocampus	[135]
*mut*-p53 increased	L	Glioblastoma	[133]
WT-p53 increased	S, K	Mouse Aorta	[136]
WT-p53 increased	D	Glioblastoma Cell lines	[131]
*mut*-p53 increased	L	Non-small cell lung cancer	[134]

**Table 2:** Prognostic and diagnostic analysis of p53 and cathepsin gene expression. Patient samples were evaluated for p53 and cathepsin expression using immunohistochemistry or flow cytometry analysis in a variety of cancer types and statistically assessed for correlations in patient survival. +D (increase cathepsin D expression); −D (decreased cathepsin D expression); % (percentage of total patients); LAC (Lung Adenocarcinoma); SSCL (Small Cell Carcinoma of the Lung); SSC (Squamous Cell Carcinoma); IBC (Inflammatory Breast Cancer); PAC (Pregnancy Associated Breast Carcinoma); BC (Breast Cancer); OC (Ovarian Cancer); CRC (Colorectal Cancer); LMaP (Low Malignancy Phenotype); Inv (Invasive).

p53 Positive	Cathepsin Positive	Study Size	Cancer Type	Reference	Survival
	+D (47%)	152	LAC	[137]	poor
	−B (23%) −D (87%)	28	SCCL	[138]	prolonged prolonged
57.8%	+D (31.25%) +D (37.50%)	64	SCC larynx	[140]	high risk of relapse
70%	+D (30%)	40	IBC	[125]	poor
46%	+D (49%)	154	SCC Oesphageal	[70]	Invasiveness/ poor prognosis
yes	+D (24.40%)	134	BC	[143]	poor
yes	+D (65.10%) LMaP +D (43.70%) Inv	43 80	OC	[145]	limited
60.9%	+D (38.70%)	266	CRC	[146]	poor
	+V (27.44%) +D (58.70%) +B (76.76%)	164 155 142	BC	[147]	poor poor
yes	+D	217	BC	[148]	relapse

**Table 3:** Cathepsin genes encode p53 half-sequences. The BAER database was scrutinized and the above cathepsin genes were identified as containing p53 consensus (or non-consensus) binding sequences for activated p53. The chromosomal (Chr) and genetic locations of these sequences are shown, as are the NCBI transcript accession numbers. Results obtained for the negative-controlled binding of p53 (in the absence of activating p53) are shown in blue (based on the availability of data).

Cathepsin	Consensus	Location	Chr	Start	End	Transcript (s)
A	no	Promoer	chr20	44519281	44519481	NM_000308,NM_001127695,NM_001167594;NM_080608
	no	Promoter	chr20	44519541	44519741	NM_000308,NM_001127695,NM_001167594;NM_080608
	no	Promoter	chr20	44518781	44518981	NM_000308,NM_001127695,NM_001167594;NM_080608
B	yes	1stExonIntron	chr8	11711471	11711751	NM_001908
	no	1stExonIntron	chr8	11720091	11720291	NM_147781,NM_147783,NM_001908
	no	1stExonIntron	chr8	11724891	11725091	NM_147781,NM_147783,NM_147780,NM_147782,NM_001908
	no	1stExonIntron	chr8	11725391	11725591	NM_147781,NM_147783,NM_147780,NM_147782,NM_001908
C	no	Intragenic	chr11	88060751	88060951	NM_001814,NM_001114173,NM_148170
	no	Promoter	chr11	88070831	88071031	NM_001114173,NM_001814,NM_148170
	no	Promoter	chr11	88070771	88071121	NM_001114173,NM_001814,NM_148170
D	yes	Intragenic	chr11	1778621	1778821	NM_001909
	no	1stExonIntron	chr11	1783701	1783901	NM_001909
	no	Intragenic	chr11	1779141	1779341	NM_001909
G	no	1stExonIntron	chr14	25044751	25044951	NM_001911
H	no	Intragenic	chr15	79222291	79222491	NM_004390
	no	Intragenic	chr15	79223191	79223541	NM_004390
	no	Promoter	chr15	79237871	79238071	NM_004390
	no	Promoter	chr15	79238121	79238321	NM_004390
	no	Promoter	chr15	79241991	79242191	NM_004390
L	no	Promoter	chr9	90340861	90341061	NM_001257971,NM_001257972,NM_001257973,NM_001912,NM_145918
	no	1stExonIntron	chr9	90341261	90341461	NM_001257971,NM_001257972,NM_001257973,NM_001912,NM_145918
O	no	1stExonIntron	chr4	156874401	156874601	NM_001334
S	yes	Intragenic	chr1	150720361	150720591	NM_001199739,NM_004079
	no	1stExonIntron	chr1	150738161	150738361	NM_001199739,NM_004079

**Table 4:** Cathepsin proteases contain predicted p53 half-sequences with the upstream 3 kb regions of their transcription start sites. The 5′ -3 kb regions for the shown cathepsin genes were taken from the Ensembl database using the show accession numbers (in italics), and a standard Matinspector search was performed to identify the location of the p53 consensus sites encoded on the positive or negative DNA strand.

Cathepsin	Start	End	Strand	Sequence	Cathepsin	Start	End	Strand	Sequence
B	1386	1410	+	aggcgggagtacaggCATGtctctg	H	2520	2544	+	ggagCGAGgtggggacaggcaggga
*CCDS5986*	1395	1419	−	gcctgtcttcagagaCATGcctgta	*CCDS10308*	2552	2576	+	ggtgCAAGgtgaagacaggcaggga
	2136	2160	+	gaatacaactggggtCATGcctgct		2562	2586	+	gaagacaggcagggaCATGgtgtga
C	353	377	+	ctaaCAAAttagctacaagattaga		2571	2595	−	gtccccacctcacacCATGtccctg
*CCDS8282*	1967	1991	−	tactaggtgtcaggcCCTGtggaca		2589	2613	−	cccctcatgtcccttCCTGtcccca
	2280	2304	−	tctggcacgcacacaCATGgcgctc		2590	2614	+	ggggaCAGGaagggacatgaggggt
	2281	2305	+	agcgcCATGtgtgtgcgtgccagag	K	2182	2206	+	gagaagctcatgtgaCTTGtcctag
D	5	29	+	ggtgcCAGGtccaggctggccgtgg	*CCDS969*	2191	2215	−	atccccaatctaggaCAAGtcacat
*CCDS7725*	506	530	−	acaaatcatttaaggCAGGtccaag	L	92	116	−	caccaggaggggtggCATGttcacc
	784	808	+	gtgcCACGttggagacaggcctcca	*CCDS6675*	1353	1377	+	gagttCAAGaccagtctggtcaata
	943	967	+	cacattggagatgggCAAGtctggg		2172	2196	+	atctCAAGagaacgacttggttacc
	952	976	−	ctcccttagcccagaCTTGcccatc	O	2469	2493	−	agccaCCTGgcctgccctgtgagcc
	1378	1402	−	taaaaccaggccgggCATGgtgact	*CCDS3794*				
	1379	1403	+	gtcacCATGcccggcctggttttac	S	248	272	−	actcaCTTGcccaggctggagtgca
	1663	1687	−	gagatggtgtttcacCATGttggcc	*CCDS968*	604	628	+	ggctacaaacacaaaCATGtctact
F	266	290	+	tttttgaaacagggtCTTGccctgt		613	637	−	ctcagctgtagtagaCATGtttgtg
*CCDS8144*	436	460	+	gaaatggggttttgcCATGttgccc		1132	1156	−	ccacgtatggtaaggCAAGtcatct
	525	549	−	ctgggcatggtggctCATGcctata		1788	1812	−	accatCTTGgccaggctggtcttga
	526	550	+	ataggcatgagccacCATGcccagc		1846	1870	−	acaggcacctgccagCATGtccagc
	617	641	−	gaagtggaagttaggCATGtttcat		2402	2426	+	ttaaaagcagtaagaCAGGttttcc
	1040	1064	−	tgtggcatggcaggtCTTGtgtcag		2658	2666	+	aCTACaagc
	1041	1065	+	tgacacaagacctgcCATGccacac		2660	2684	−	ctgggcatggtggtgCATGcttgta
	1552	1576	+	aaagttaccttggccCATGcccagg		2948	2972	+	gagtacctcatgtgaCAAGttccaa
	1562	1586	+	tggccCATGcccaggaatgagtgaa	V	662	686	−	ccaggcatggtgatgCATGcctgta
	1809	1833	−	ctgggCATGgtggcacgtgcctgta	*CCDS6723*	663	687	+	acaggcatgcatcacCATGcctggc
	1810	1834	+	acaggcacgtgccacCATGcccagc		1377	1399	−	gaATAAtatccacagtttttact
H	1370	1394	−	caccttgcaaagtggCATGttgttg	W	246	270	−	acctCAAGcaatccacctgccttgg
*CCDS10308*	1824	1848	+	ggtgCAAGgtggagacacgcaggga	*CCDS8117*	773	797	−	tgggccccagccagtCTTGtcctgt
	1833	1857	−	cccctcatgtccctgCGTGtctcca		1486	1506	+	agcccttgACCTcacaagtca
	1834	1858	+	ggagacacgcagggaCATGaggggg		1945	1969	−	aaaaCAAAaccaggccaggcacggt
	1889	1913	+	ggtgCAAGgcggggacaggcaggga		2301	2325	−	gctaggcaTGAGtcaggctcgctag
	1993	2017	−	acccccatgtctctgCCTGtcccca		2671	2695	−	acaggcatgcaccacCATGcccagc
	1994	2018	+	ggggacaggcagagaCATGggggtg		2672	2696	+	ctgggcatggtggtgCATGcctgta
	2116	2140	−	ccccccatgtccctgCCTGtctcca	Z	102	126	+	cgcccccacaaggaaCATGtttaag
	2117	2141	+	ggagacaggcagggaCATGgggggt	*CCDS13474*	111	135	−	atgcaagatcttaaaCATGttcctt
	2180	2204	−	ctccccatgtccctgCTTGtcccca		276	300	−	ctagtcgagtggatgCATGtctggc
	2181	2205	+	ggggacaagcagggaCATGgggagt		1227	1251	−	tcagCAAGgcaggcacacgacccct
	2212	2236	−	cgccccacgtccctgCCTGtcccca		1786	1810	+	caccgccagctcaagCTTGgggact
	2275	2299	−	ctccccatgtccctgCCTGtctcca		1872	1896	−	ctcttatttgtttggCAAGtcgctc
	2276	2300	+	ggagacaggcagggaCATGgggagt					
	2404	2428	+	ggggacaggcagggaCATGggggtg					
